# Peer Teasing and Restrained Eating among Chinese College Students: The Chain Mediating Role of Negative Coping Styles and Negative Physical Self

**DOI:** 10.3390/nu16010163

**Published:** 2024-01-04

**Authors:** Yuwansu Wang, Yijun Luo, Jie Zhao, Yicen Cui, Hong Chen

**Affiliations:** 1 Faculty of Psychology, Southwest University, Chongqing 400715, China; wsu999@email.swu.edu.cn (Y.W.); luoyijun@swu.edu.cn (Y.L.); zj2000@email.swu.edu.cn (J.Z.); littlecui97@email.swu.edu.cn (Y.C.); 2 Key Laboratory of Cognition and Personality (SWU), Ministry of Education, Chongqing 400715, China; 3 China Research Center of Psychology and Social Development, Chongqing 400715, China

**Keywords:** peer teasing, restrained eating, negative coping styles, negative physical self

## Abstract

This study aimed to determine whether negative coping styles and negative physical self sequentially mediate the relationship between peer teasing and restrained eating among Chinese university students. In total, 1127 participants (66.9% women, average age = 18.43 years; age range, 14–26 years) completed the Perception of Teasing Scale, Coping Style Questionnaire, Negative Physical Self Scale, and the Chinese version of the Restraint Scale. The mediational analysis showed that, after controlling for age, sex, and body mass index (BMI), peer teasing was related to restrained eating behaviors through (a) the mediating effect of negative coping styles, (b) the mediating effect of negative physical self, and (c) the chain-mediating effect of negative coping styles and negative physical self. This study showed for the first time that negative coping styles and negative physical self may chain mediate the association between peer teasing and restrained eating. It also provides suggestions for clinical practices as to strategies for controlling restrained eating.

## 1. Introduction

The term restrained eating (i.e., controlling food intake to alter external appearance) refers to eating behaviors that have negative effects on an individual’s physical and mental well-being. These behaviors have become hot topics in health psychology [1]. A study conducted on >80,000 adolescents found that 28% and 56% of boys and girls, respectively, reported engaging in one or more negative eating behaviors [2]. On 10 May 2023, the China University of Social Sciences and Social Science Literature Publishing House jointly released a research report titled “Study on Health Behaviors of Chinese Adolescents—Analysis of Survey Data from 13 Provinces”. The study indicates that most adolescents are not very satisfied with their body weight, and 9.2% of adolescents choose to engage in restrained eating for weight loss. Since restrained eating behaviors can have serious and negative consequences on physical (e.g., nutrient imbalance, insufficient energy intake, and anemia) and mental health (e.g., decreased self-esteem and depression) [3,4], it is critical to be aware of the factors contributing to restrained eating.

Previous studies on diet have found that negative peer relationships, such as social pressure regarding appearance and verbal bullying from peers, can lead to adolescents engaging in restrained eating as a coping mechanism with these negative influences [5,6]. Research has internationally shown that bullying (including ridicule) by peers is a common phenomenon for individuals learning in a school setting [7], and it occurs chronically and repeatedly [8]. Peer teasing is a major form of bullying [9], and is the negative evaluation of verbal abuse and taunting by peers related to physical appearance and behavioral ability [10]. Among adolescents, peer relationships become increasingly important, and recent research has found that peer teasing can lead to unhealthy eating behaviors [11]. Furthermore, teasing peers is a direct factor contributing to eating disorders [12]. Regarding the potential benefits of peer interactions for eating behavior, a past meta-analysis showed that positive peer influence was associated with less unhealthy eating behavior [13], and a study depicted that peer relationships in the campus system are important in daily life and serve as a significant source for the development of various social skills and behavioral choices [14]. These findings on the benefits of positive peer influence and the potentially detrimental effects of negative peer relationships collectively imply that peer ridicule about one’s body can further lead individuals to internalize this kind of body discrimination [15]. Furthermore, peer teasing about the body can lead individuals to change their eating behaviors to achieve a “standard” body to avoid ridicule [16]. Regarding the relevance of sex for related discussions, while peer evaluations may have had a greater impact on girls’ dieting behavior (vs. boys) in the past—leading them to adopt negative eating practices to achieve a “slim figure” [17]—boys, too, nowadays may often be impacted by peer evaluations and try to modify their physique by reducing their carbohydrate intake [18].

Although the impact of peer teasing on the development of restrained eating has been confirmed [19], the exploration of its internal mechanisms remains quite limited. One variable that may be related to this impact is the negative physical self. As an important research topic in the field of body image, a negative physical self refers to individuals’ negative perception, negative emotional experiences, and corresponding behavioral regulation regarding their own physical appearance, including aspects such as weight, height, and facial features [20]. Research shows that individuals start incorporating peer evaluations into their self-system ever since childhood, and that peer teasing during childhood influences the relationship between weight status and self-concept [21]. Then, as individuals progress into college, peer evaluations become increasingly important and a significant component of self-concept [22]. The increased significance given to peer evaluations may lead individuals to experience dissatisfaction with their bodies after receiving negative evaluations from their peers [23]. Chen and Jackson conducted a longitudinal study and found that peer teasing played a significant role in the formation of negative self-body image [24]. Furthermore, there is accumulating evidence suggesting that the negative physical self is a proximal factor implicated in various disordered eating behaviors among college students [25,26,27]. The impact of negative physical self on eating disorders tends to intensify over time [28] and can predict eating disorder severity [27]. Therefore, we may infer that the negative physical self can mediate the effect of peer teasing on restrained eating.

There is also some evidence pointing to the possibility of coping style serving as a mediator in the relation of peer ridicule and restrictive eating. Coping style is the primary way through which college students deal with stressful events, referring to a strategy that determines cognitive and behavioral outcomes when facing stressors [29]. Coping styles typically include two types: active coping (e.g., seeking problem solving and help) and passive coping (e.g., self-blame, fantasy, forgiveness, avoidance, and rationalization) [30]. The choice of coping style depends on the nature of specific events, often leaning towards passive coping after experiencing negative life events such as lack of peer support and negative parenting [31,32]. According to Selman’s perspective on the development stages of social perspective taking, normal peer interactions provide an important background for individuals to learn social behaviors, such as seeking help and respect, and adaptive abilities. Therefore, research suggests that peer ridicule, as a negative event in peer interactions, can lead individuals to adopt passive coping styles to distract attention or provide a potential avoidance strategy, which may further exacerbate psychological and behavioral problems [33,34]. Meanwhile, other studies indicate that the adoption of passive coping strategies such as giving up, denial, and self-blame when dealing with peer-induced stressors may, to some extent, serve as a short-term and effective self-protective strategy [35,36]. Additionally, existing research found that the adoption of passive avoidance coping styles may lead to more severe restrictive eating behaviors, which may be influenced more by negative stimuli than cognitive control. According to the attentional failure theory, the use of avoidance coping may weaken the cognitive processing of negative events and further intensify restrictive eating behaviors [37,38]. The predominance of passive coping strategies characterized by avoidance may lead individuals to continuously internalize stressful events, and peer ridicule related to abilities and appearance may lead individuals to engage in restrictive eating as a means of changing peer evaluation and seeking peer approval [39,40]. Therefore, it is reasonable to hypothesize that negative coping styles may mediate the effect of peer ridicule on restrictive eating behaviors.

Passive coping styles are positively correlated with negative body image [41]. According to the theory of early maladaptive schemas, the belief in self-defeat regarding oneself, others, and the world, formed by the accumulation of negative experiences from negative life events, leads to more negative self-cognition [42]. Longitudinal evidence suggests that positive coping styles can alleviate postoperative negative body image in breast cancer patients, whereas avoidance, fear, and other negative coping styles may exacerbate patients’ negative body self-assessment [43]. This evidence supports this study’s viewpoint that the connection between negative coping styles and negative body image may be associated with the adoption of restrictive eating behaviors.

In summary, this study proposes the following hypotheses: (1) there is a significant correlation between peer teasing and restrained eating; (2) negative coping styles mediate the relation between peer teasing and restrained eating; (3) negative physical self mediates the relation between peer teasing and restrained eating; and (4) negative coping styles and negative physical self play a chain-mediating role in the relation between peer teasing and restrained eating. To this end, this study attempted to develop a chain-mediation model to explore these variable relations. The evidence of this research is expected to provide knowledge on potential associations that can be targeted by interventions for reducing peer teasing and overcoming restrained eating, which may then support the promotion of college students’ physical and mental development.

## 2. Materials and Methods

### 2.1. Participants

The data were collected from our ongoing project named the Behavioral Brain Research Project of Chinese Personality (BBP). It employs convenience sampling to recruit college students from various departments of a university in Chongqing, China, and then uses a random assignment method for participant allocation post sampling. Before participants undergo data collection procedures (i.e., behavioral variable measurements), they are screened for self-reported medical illness, neurological illness, and psychiatric disorders (i.e., through binary self-reported variables indicating the presence or absence of particular diseases, disorders, and use of psychoactive medications). The participants are then requested to respond to each question as honestly as possible to minimize the influence of social desirability bias.

**Figure 1:**
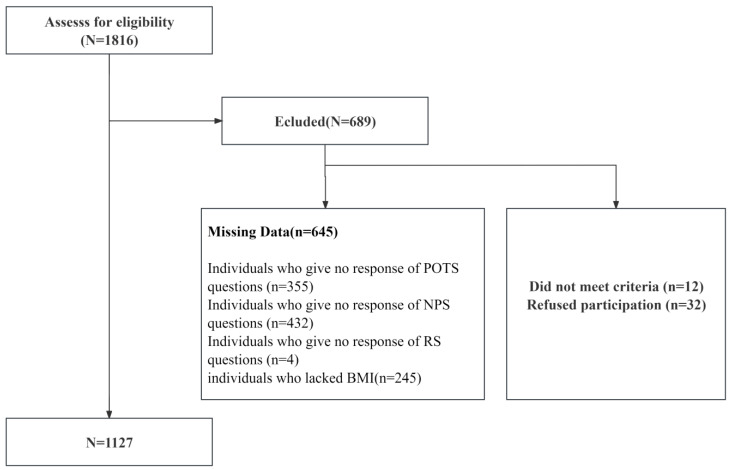
Flow chart of the screening process for the selection of eligible participants.

All participants of this study signed an informed consent form before participation in the study and received an honorarium of CNY 5 (approximately USD 0.77) upon completion of their participation. Ethical approval for this study was granted by the Ethics Committee Board of the university to which this study is related. Prior to screening, the study sample comprised 1816 participants (Figure 1).

A final sample of 1127 participants (754 females) ranging from 15–26 years (M = 18.43, SD = 1.04) was included in the analysis. Their mean age was 19.8 (SD = 1.75), with an age range of 15–26. Their mean body mass index (BMI) was 21.39 (SD = 3.07), with a BMI range of 10.24–35.54, and sample characteristics are shown in Table 1.

### 2.2. Measures

#### 2.2.1. Perception of Teasing Scale

The Perception of Teasing Scale (POTS), developed by Thompson et al. [44], was used to describe the frequency of peer teasing and the degree of being affected by peer teasing. The POTS consists of 11 items in total, and the content of ridicule includes both physical and ability ridicule; the entire questionnaire is divided into two subscales of ridicule content and impact. Each item is scored on a 5-point Likert scale, with the frequency of peer ridicule ranging from 1 (never) to 5 (very frequent) and the degree of being affected by peer ridicule ranging from 1 (never frustrated) to 5 (very frustrated). Higher scores indicate higher levels of peer ridicule and influence on individuals. In this study, the Cronbach coefficient of this scale was 0.94.

#### 2.2.2. Coping Style

The simple coping style questionnaire developed by Jie [45] was used to describe individuals’ attitudes toward and ways of coping with life events. The scale consists of 20 questions and includes two dimensions: positive and negative coping styles. The present study adopted the subscale of negative coping style. Items are rated on a 4-point Likert scale from 0 (not taken) to 3 (often taken). The higher the subscale score, the more pronounced the adoption of a negative coping style. In this study, the Cronbach coefficient of this scale was 0.70.

#### 2.2.3. Negative Physical Self

Chen developed the Negative Physical Self Scale (NPSS) [46] to describe negative perceptions, negative affective experiences, and corresponding behavioral modifications regarding one’s body. The scale comprises 48 items and includes 5 dimensions: overall, fat, thin, short, and appearance. The items are scored on a 5-point Likert scale ranging from 0 (never) to 4 (always). Higher scores indicate that respondents are more dissatisfied with their own bodies. In this study, the Cronbach coefficient of this scale was 0.89.

#### 2.2.4. Restrained Eating

The Chinese version of the Restraint Scale (RS) revised by Kong et al. [47] was used. The scale contains 10 items measuring both dietary concerns and weight change, with questions 5–8 scored on a 4-point Likert scale from 0 (never) to 3 (always), and questions 1–4 and 9–10 on a 5-point scale, with higher scores indicating higher levels of restrictive eating. In this study, the Cronbach coefficient of this scale was 0.75.

#### 2.2.5. Control Variables

Considering that age, sex, and BMI are all important factors associated with restrained eating, these variables were treated as covariates. Age, sex, weight, and height were self-reported. The BMI was calculated using the standard formula of weight (kilograms) divided by height (meters) squared (BMI = kg/m^2^).

### 2.3. Data Analysis

The data were organized and analyzed using SPSS 26.0. IBM SPSS Statistics is a software product of IBM Corporation, headquartered in Armonk, New York, United States. It was first used to count variable distribution and calculate variable correlation. Bootstrap analysis [48] with 5000 iterations was conducted using PROCESS Macro to assess the effects of peer teasing on restrained eating through the mediation of negative coping styles and negative physical self.

## 3. Results

### 3.1. Common Method Biases Test

In view of the possible common method bias from the use of self-reporting data collection methods, the necessary procedural controls, such as protecting respondents’ anonymity, explaining to participants that the use of the data obtained would be restrained to scientific research, and using reverse presentation for some items, were conducted during the administration process. To further improve study rigor, Harman’s one-way test (i.e., unrotated factor analysis for all variable items) was used as a statistical control before formal data analysis. The results showed that the variance explained by the first factor was 16.77%, which is less than the threshold of 40%. Therefore, no significant common method bias was observed in the data used in this study.

### 3.2. Preliminary Analyses

The descriptive statistics and correlation matrix for the study variables are presented in Table 2.

### 3.3. The Chain-Mediation Analyses

**Figure 2:**
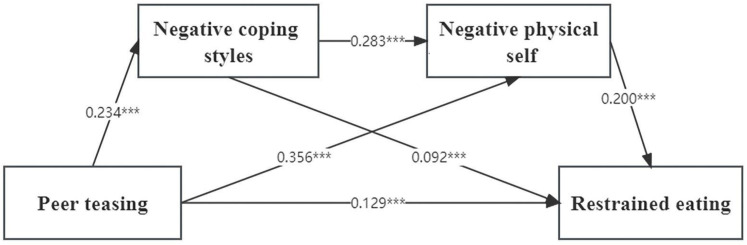
Serial mediation model testing negative physical self and restrained eating as mediators between peer teasing and negative coping style. Standardized coefficients, *** *p* < 0.001.

Table 3 shows the mediation results. The path coefficient of the total effect of peer teasing on restrained eating was significant (*p* < 0.001). Next, the mediating variables, negative coping style, and negative physical self, were added to the model to obtain the path model shown in Figure 1. The results showed that all paths reached a statistically significant level (*p* < 0.001). The model not only showed the direct effect of peer teasing on restrained eating but also that negative coping style and negative physical self played a mediating role in the relationship between peer teasing and restrained eating. There were three pathways, as follows: at first, we observed a separate mediating role of negative coping style and a separate mediating role of negative physical self; bootstrap results showed that negative coping style, negative physical self, and negative coping style–negative physical self had mediating effects. Figure 2 illustrates the serial mediation model.

The direct effect of peer teasing on restrained eating was 0.1764, with a confidence interval of [0.0973, 0.2554]. Furthermore, X had a significant indirect influence on Y, with an effect size of 0.7735 and a confidence interval of [0.6808, 0.8620]. Among the mediating variables, the effect size obtained when using M1 as the mediator was 0.5298, with a confidence interval of [0.4468, 0.6100]. The effect size obtained when using M2 as the mediator was 0.1240, with a confidence interval of [0.0790, 0.1725]. The effect size obtained when both M1 and M2 were used as serial mediators was 0.1197, with a confidence interval of [0.0753, 0.1685]. The effect sizes of the three mediation paths accounted for 55.774%, 13.430%, and 12.601%, respectively. All the effect sizes of the models fell within their respective confidence intervals, which did not include zero.

## 4. Discussion

This study examines the association between peer teasing and restrained eating among Chinese college students. The mediating effects of negative coping style and negative physical self on this association were also investigated. Our data demonstrates that peer teasing is associated positively with restrained eating. Consistent with previous studies [5,28], our results confirmed this association to be mediated by negative physical self. Additionally, we showed for the first time that peer teasing could be associated with restrained eating through (a) the mediating effect of negative coping style and (b) the chain mediating effect of negative coping style and negative physical self.

As predicted, the negative physical self mediated the association between peer teasing and restrained eating among Chinese college students. For example, Rodgers et al. [49] found that peer teasing in early adolescence positively correlated with unhealthy weight control behaviors about restrained eating, and this effect was directly mediated by a negative body image. Similarly, Duarte et al. [28] found that a negative physical self was a significant mediator in the relationship between peer teasing and restrained eating. According to the tripartite influence model proposed by Thompson et al. [50], when influenced by external pressures (e.g., peer pressure), college students may experience pressure to conform to specific appearance standards. When unable to meet these standards, they may develop a negative body image. The notion of a negative physical self refers to a form of “perceiving a flaw in oneself” that can lead individuals to engage in certain behaviors in an attempt to compensate for this “flaw”, which directly predicts restrictive eating behavior [24]. Therefore, the focus of prevention and treatment efforts for restrictive eating could be placed on fostering critical thinking skills or positive self-awareness [27,33].

This study also examined the mediating role of negative coping styles in the relationship between peer teasing and restrained eating. Because of the psychological reasons underlying coping strategy choice, the consequences of their use, and the importance of coping strategies in college students’ management of stress and handling of life events, coping styles have attracted extensive attention from researchers. Previous studies have found associations between negative coping styles and negative interpersonal events [31,36,51], negative body image [41,42,43], and disordered eating behavior [37,38,39]. College students use coping strategies as the main approach for dealing with stress, interpersonal adaptation, and conflict regulation [31]. Negative coping styles are typically triggered by negative life events [31,32,36] and subsequently lead to various negative cognitions [43,44] and behaviors [41,42]. Previous studies showcase that coping strategy choice is directly related to perceived environmental support, and the nature of stressful events and can lead to different behavioral outcomes [51]. According to social–cultural theory, peer relationships and social interactions play a central role in campus interpersonal relationships, help college students perceive social support, and contribute to self-awareness development. However, recent research has found that peer ridicule is a negative event within peer relationships that can undermine perceived social support among individuals [13,16]; in such situations, college students become more likely to respond with passive avoidance and negative coping strategies [52], which can help, to some extent, individuals temporarily escape the pressure brought by peer events. It can be perceived as a temporary self-protective measure. However, negative evaluations from others, when accompanied by the absence of timely reassessment and self-affirmation of own worth [19], can have negative impacts that lead to severe psychological problems and further behavioral issues [12,23]. Consequently, in the long term, there may be an increased risk of engaging in other harmful behaviors, such as restrictive dieting.

These discussions show that coping styles may come into play between peer teasing and restrained dieting owing to their roles as primary adaptive strategies among college students. In today’s campus life, the interpersonal adaptation skills and stress event management abilities of college students permeate all aspects of their campus lives, directly influencing their interpersonal experiences and overall life satisfaction. Issues related to diet may require stronger personal control compared to other behavioral problems [53]. Therefore, proactive coping strategies may have a positive relationship with the avoidance of eating disorders, whereas negative coping strategies (e.g., avoidance) may intensify the perception of distress and a sense of loss of control, making it easier for individuals to choose extreme problem-solving methods [31,53]. For example, adopting self-blame or self-criticism as coping strategies when faced with peer ridicule can encourage individuals to resort to extreme dieting behaviors to address the non-acceptance of their appearance. This, in turn, leads to various maladaptive adaptations and psychological issues. To support these assumptions, a systematic review has demonstrated that coping strategies directly influence various future risk behaviors, including eating disorders [54]. Therefore, supporting the adoption of more positive coping strategies, such as humor and positive interpretations of others’ evaluations, can be associated with the prevention of various psychological and behavioral issues [55]. It may also be related to more positive dietary behaviors and the promotion of self-health development.

Our data further indicated that a negative coping style could indirectly influence restrained eating through negative physical self; that is, a negative coping style may be associated with the strengthening of the internalization of peer teasing content. The theory of negative self-schema further emphasizes this relationship, as excessive negative responses to others’ evaluations can exacerbate dissatisfaction with their own body, leading to a stereotyped and emotional reaction to body-related information (e.g., self-weight and body shape) and an excessive emphasis on outward self-representation [56]. Studies have depicted that the external environment in which one grows up influences individual attitudes toward things and has a certain impact on development [57]. Therefore, peer teasing may be related to an intensification of negative social adaptation and the deepening of individual negative self-evaluation; meanwhile, a negative body self-perception may be associated with the mismanagement of negative response styles to peer ridicule. In summary, college students may choose to adopt restrained eating behaviors in relation to attempts to cope with the negative perception of their bodies.

To summarize, our study has extended the scope of previous research by revealing the novel mediating role of negative coping styles and the chain-mediating role of negative coping styles and negative physical self in the relationship between peer teasing and restrained eating among Chinese college students. These findings have important clinical implications for the prevention and treatment of restrained eating and insights for intervention research on college students’ restrictive eating behaviors. For example, college students may come to focus on their psychological and physical health in campus life. When facing peer teasing, the findings of this study suggest that there may be a correlation between students being able to deal better with the situation and them engaging in the following behaviors: being firm in self-awareness; accepting reasonable suggestions from others; taking the initiative to maintain self-independence and uniqueness; addressing negative comments or stressful events from others through proactive and positive behaviors; resolving difficulties after a rational analysis of the events without compromising their physical and mental well-being. Therefore, it may be important for stakeholders to endeavor to prevent peer ridicule behaviors that occur in school or daily life, actively devise strategies to promote self-confidence, -esteem, and -love, and develop a healthy understanding of the importance of positive eating behaviors.

Although this study expanded existing research on the effects of restrained eating to some extent, there are some limitations that need to be addressed in future studies. First, despite this study and its examinations being based on past research and theories, its cross-sectional design hinders our ability to provide causality inferences. Future experimental and longitudinal research could explore in-depth the influence mechanisms between the variables we explored. Second, the effect sizes we observed were small to moderate, and thus generalizations should be made with caution; still, the study sample was relatively large, allowing for significant results to be obtained even with small effect sizes. Finally, this study used mostly self-reports from college student groups who may be more concerned and confronted with peer influences in their lives, including language and behavior, and therefore have more influence on restrained eating. Future research should explore the influence of peer teasing on the restrained eating of other groups.

## 5. Conclusions

This study depicts that for Chinese college students, peer teasing is significantly and positively associated with restrained eating. Moreover, negative coping styles and negative physical self mediated the relationship between peer teasing and restrained eating through three pathways: the mediation of negative coping styles, the mediation of negative physical self, and the chain mediation of negative coping styles–negative physical self. Future research should explore the influence of peer teasing on restrained eating in other groups.

## Figures and Tables

**Table 1:** Sample characteristics.

Characteristics	Options	Frequency	Percentage (%)
1. Sex	Male	373	33.2
	Female	751	66.8
2. Age	14–17	54	4.8
	17–20	1001	88.8
	20–23	69	6.1
	23–26	3	0.3
	<17.49 kg/m^2^	72	6.4
3. BMI	17.5–23.99 kg/m^2^	853	75.7
	24–27.99 kg/m^2^	167	14.8
	≥30 kg/m^2^	35	3.1

**Table 2:** Correlations, means, and standard deviations of study variables.

Variables	M (SD)	Correlations					
		1. Age	2. BMI	3. PT	4. NCS	5. NPS	6. RE
1. Age	18.43 (1.05)	—					
2. BMI	21.39 (3.07)	−0.10 **	—				
3. PT	38.72 (17.58)	−0.10 ***	0.16 ***	—			
4. NCS	10.46 (4.05)	0.04	0.08 **	0.24 ***	—		
5. NPS	114.21 (22.50)	0.07 *	0.22 ***	0.42 ***	0.28 ***	—	
6. RE	12.25 (5.85)	0.00	0.41 ***	0.32 ***	0.20 ***	0.37 ***	—

Note: BMI = body mass index; M = mean; NCS = negative coping styles; NPS = negative physical self; PT = peer teasing; RE = restrained eating; SD = standard deviation. *** *p* < 0.001, ** *p* < 0.01, * *p* < 0.05.

**Table 3:** Mediating effect analysis of the chain-mediating model path.

Path	Mediating Effect Value	Standard Error	Lower Limit of 95% CI	Upper Limit of 95% CI	*p*
Total effect	0.230	0.025	0.182	0.278	<0.001
Direct effects	0.130	0.026	0.078	0.180	<0.001
Total mediation effect	0.101	0.014	0.075	0.129	<0.001
Peer teasing → Negative coping style → Restrained eating	0.022	0.001	0.010	0.035	<0.001
Peer teasing → Negative physical self → Restrained eating	0.071	0.012	0.048	0.096	<0.001
Peer teasing → Negative coping style → Negative physical self → Restrained eating	0.009	0.002	0.005	0.014	<0.001

## Data Availability

To promote data transparency, anonymized data that support the findings of this study are available from the corresponding author upon reasonable request. The data are not publicly available due to these data are derived from the Behavioral Brain Research Project of Chinese Personality (BBP).
